# Materia Medica and Therapeutics

**Published:** 1884-09

**Authors:** 


**Materia Medica and Therapeutics.** An Introduction to the Rational Treatment of Disease. By J. M. BRUCE, M. A., M. D., Lecturer on Materia Medica and Therapeutics, Charing Cross Hospital, London. Philadelphia: H. C. Lea's Son & Co.

